# Identification of Conflicting Selective Effects on Highly Expressed Genes

**Published:** 2007-02-14

**Authors:** Paul G. Higgs, Weilong Hao, G. Brian Golding

**Affiliations:** 1 Department of Physics and Astronomy, McMaster University, Hamilton, Ontario L8S 4M1.; 2 Department of Biology, McMaster University, Hamilton, Ontario L8S 4K1.

**Keywords:** Translational efficiency, Translational robustness, Codon Usage, Amino Acid Usage, *Saccharomyces cerevisiae*

## Abstract

Many different selective effects on DNA and proteins influence the frequency of codons and amino acids in coding sequences. Selection is often stronger on highly expressed genes. Hence, by comparing high- and low-expression genes it is possible to distinguish the factors that are selected by evolution. It has been proposed that highly expressed genes should (i) preferentially use codons matching abundant tRNAs (translational efficiency), (ii) preferentially use amino acids with low cost of synthesis, (iii) be under stronger selection to maintain the required amino acid content, and (iv) be selected for translational robustness. These effects act simultaneously and can be contradictory. We develop a model that combines these factors, and use Akaike’s Information Criterion for model selection. We consider pairs of paralogues that arose by whole-genome duplication in *Saccharmyces cerevisiae.* A codon-based model is used that includes asymmetric effects due to selection on highly expressed genes. The largest effect is translational efficiency, which is found to strongly influence synonymous, but not non-synonymous rates. Minimization of the cost of amino acid synthesis is implicated. However, when a more general measure of selection for amino acid usage is used, the cost minimization effect becomes redundant. Small effects that we attribute to selection for translational robustness can be identified as an improvement in the model fit on top of the effects of translational efficiency and amino acid usage.

## Introduction

The genome of *S. cerevisiae* retains many pairs of paralogous genes that arose by whole-genome duplication (Kellis et al. 2004). These pairs of paralogues provide a large set of comparable genes that have been evolving and diverging subject to the same selective forces for the same amount of time. They are thus an ideal data set on which to test some of the fundamental theories about how selection acts on gene sequences. We begin by considering several well documented effects. Firstly, selection for efficiency of translation is an important factor causing biased codon usage in *S. cerevisiae* (Percudani et al. 1997; Akashi, 2003), *Drosophila* (Powell and Moriyama, 1997), humans (Comeron, 2006) and other species (Chen et al. 2004; Rocha, 2004). Preferred codons are those for which the tRNAs are most abundant, and for which the gene copy number is largest. Codon bias is observed to increase as a function of expression level, suggesting that translational efficiency is most important in highly expressed genes. Although translational selection is usually considered as selection for increasing the speed of translation, selection for translational accuracy may also play a role (Akashi, 1994).

Secondly, amino acid composition also varies with expression level. It has been argued that amino acids with high total tRNA abundance are preferred in high-expression genes (Lobry and Gautier, 1994; Akashi, 2003), i.e. translational efficiency influences amino acid composition as well as codon usage. It has also been found that selection can act to minimize the energetic costs of amino acid synthesis. As a result, highly expressed genes have higher frequencies of the least costly amino acids (Akashi and Gojobori, 2002; Jansen and Gerstein, 2000).

Thirdly, in addition to the selective effects above, amino acid composition of proteins is also influenced by mutation pressure arising from the unequal base frequencies in the DNA (Knight et al. 2001; Bharanidharan et al. 2004; Urbina et al. 2006). If the GC content is high or low, this favours the amino acids whose codons have high or low GC content. We show below that in high-expression genes, selection on amino acid usage causes amino acid frequencies to differ more from the expectations based on GC content than in low-expression genes. A similar effect was seen in pseudogenes (Echols et al. 2002).

Fourthly, Drummond et al.(2005) showed that high-expression genes in *S. cerevisiae* evolve more slowly than low-expression genes, and proposed a new hypothesis of translational robustness to explain this. Translational error leads to amino acid replacements in proteins that may cause misfolding. The translational robustness theory proposes that proteins are selected to be tolerant to the effects of translational error, i.e. the amino acid sequence is selected to fold properly despite translational errors. A direct test of this hypothesis would involve an experimental study of how proteins fold when they contain amino acid substitutions. However, if the hypothesis is true, it should have the following observable consequences on the frequencies of codons and amino acids that are subject to test by sequence analysis. High-expression genes should preferentially use codons with higher numbers of synonymous neighbour codons because synonymous errors will have no effect. High-expression genes should also preferentially use codons where errors lead to replacements by amino acids with similar physical properties, because these errors should have less disruptive effects on protein structure.

The layout of the genetic code is itself optimized to reduce the effects of translational error (Haig and Hurst, 1991; Freeland and Hurst, 1998). Errors are more frequent at 1st and 3rd positions than 2nd (Parker, 1989; Freeland and Hurst, 1998), and it is found that the genetic code is arranged such that 1st and 3rd position errors cause smaller changes in physical properties than 2nd position changes. Studies on mitochondrial gene sequences clearly illustrate this positional effect (Urbina et al. 2006) and show that the variability in frequencies of bases and amino acids among species is strongly dependent on the genetic code structure. Archetti (2004, 2006) has found evidence that selection for error minimization at the protein level can influence codon usage in *Drosophila* and rodents. However, Marquez et al.(2005) did not find evidence for this effect.

There are several, potentially conflicting, selective effects on gene sequences. Thus, a statistical method is required that will allow many factors to be considered simultaneously and their relative magnitudes to be assessed. We make use of Akaike’s Information Criterion (AIC), derived from Maximum Likelihood theory, which provides a principled method of model selection (Burnham and Anderson, 1998).

## Materials and Methods

A total of 290 pairs of paralogues with expression level published in Drummond et al. (2005) were examined. The DNA sequence of each gene was extracted from the complete *S. cerevisiae* genome (Goffeau et al. 1996). The DNA sequence of each gene was translated to a protein sequence and the protein pairs were aligned using ClustalW (Thompson et al. 1994). Nucleotide sequence alignments were created from the protein alignments by replacing each amino acid with its corresponding codon. The total number of codons in this data set is *n_tot_* = 146398. Codon-based models will be used with 32 states that correspond to codon blocks translated by distinct groups of tRNAs. Each single-codon amino acid and each two-codon amino acid is a single block. Ile is two blocks: AUY and AUA. Four codon amino acids are treated as two blocks of pryimidine and purine-ending codons (e.g. Val = GUY and GUR). The 6-codon amino acids were treated as 3 blocks: Leu = UUR, CUY and CUR; Ser = UCY, UCR and AGY; Arg = CGH, CGG and AGR (where H denotes U, C or A). These blocks were based on the set of tRNA genes in the *S. cerevisiae* genome (Percudani et al. 1997) and the wobble pairing rules. The grouping of pyrimidine-ending pairs together is clear, because in every case they are translated by a single type of tRNA. Some purine-ending pairs have a single type of tRNA with U at the wobble position, but others have two tRNA types with U and C at the wobble position. It is expected that tRNAs with C at the wobble position translate only G-ending codons, but those with U translate both A- and G-ending codons. For this reason, A- and G-ending codons are not independent, so we grouped them into a single block. The Arg(CGN) family is an exception, because the two tRNA types have anti-codon ICG (translating CGU, CGC, and CGA codons) and CCG (translating only CGG). Hence, these codons were split into a block of 3 and a single-codon block.

## Results

### Causes of asymmetry

Let *n_ij_* be the number of sites at which state *i* in the low-expression gene is aligned with state *j* in the high-expression gene (states *i* and *j* are one of the 32 codon blocks defined above). Let Δ*n_ij_* = *n_ij_* − *n_ji_*. If the mutational process is symmetrical with respect to interchange of high and low expression genes, we would expect Δ*n_ij_* = 0 (plus or minus statistical variation) for every *i* and *j*. Significant deviation of Δ*n_ij_* from 0 indicates an asymmetry in the substitution process between high and low expression genes. We now propose a series of factors that might be expected to cause such an asymmetry. For each proposed asymmetry factor, we define a function δ(*i, j*) that is proportional to the predicted strength of the effect for substitutions between states *i* and *j*.

The first predicted effect is that selection for translational efficiency causes a preference for codons with a higher number of matching tRNA genes and that this preference will be stronger in high expression genes. Let *N_tRNA_* (*i*) be the number of tRNA gene copies for codon block *i* (Table 1). The increase in copy number when mutating from *i* to *j* is *δ_tRNA_* (*i, j*) = *N_tRNA_* (*j*) − *N_tRNA_*(*i*). According to this prediction, Δ*n_ij_* should be positive if the number of tRNAs for codon block *j* is larger than for *i*.

The next prediction is that selection will cause a preference for amino acids of low synthetic cost and that this preference will be stronger in high expression genes. We use two different estimators of the cost of amino acid synthesis (Table 2). *N_ATP_*(*a*) is the number of ATP molecules required for biochemical synthesis of amino acid *a* (Akashi and Gojobori, 2002) and *MW*(*a*) is the molecular weight of *a*, which has been argued to be a more general cost measure than the ATP cost (Seligmann, 2003). According to these two measures, the savings in cost due to a mutation from *i* to *j* are *δ_ATP_* (*i, j*) = *N_ATP_*(*a*(*i*)) − *N_ATP_*(*a*(*j*)) and *δ_MW_*(*i*, *j*) = *MW*(*a*(*i*)) − *MW*(*a*(*j*)), where *a*(*i*) and *a*(*j*) are the amino acids coded by blocks *i* and *j*. Note that the order of the indices *i* and *j* in these two definitions is reversed in comparison to δ*_tRNA_*(*i, j*) because the prediction is that there will be a decrease in cost but and increase in *N_tRNA_*. In each case, the sign of δ(*i, j*) is chosen so that a positive value is a predictor of a positive δ*n_ij_*.

In absence of selection, the frequency of the bases should be controlled by the equilibrium frequencies of the mutation process. If the equilibrium GC frequency is φ, the frequencies of the bases should be φ*_G_* = φ*_C_* = φ/2 and φ*_A_* = φ*_U_* = (1 − φ)/2. If a gene is under no selection other than the fact that stop codons are not used within the gene, the frequency of codon XYZ should be φ*_X_*φ*_Y_*φ*_Z_*/*S*, where *S* is the sum of φ*_X_*φ*_Y_*φ*_Z_* over all codons other than stop codons (*S* = 1− (1 + φ)(1 − φ)^2^/8). We obtain predicted amino acid frequencies by summing these codon frequencies for each amino acid. We estimate that φ = 0.3464 from the GC content at fourfold-degenerate sites. Table 2 shows the predicted and observed frequencies, *f_pred_*(*a*) and *f_ave_*(*a*). We use ‘ave’ to denote the average of observed frequencies in high- and low-expression genes. The difference Δ*f*(*a*) *= f_ave_*(*a*) − *f_pred_*(*a*) is a measure of selection on amino acid usage (AAU). This could be positive or negative, according to whether the amino acid is preferred or avoided. The next predicted asymmetry effect is that high-expression genes should be under stronger AAU selection than low-expression genes, i.e. if Δ*f* (*a*) is positive, the frequency of *a* should be higher in high expression genes than low expression genes, and *vice versa* if Δ*f* (*a*) is negative. We define δ*_AAU_*(*i, j*) = Δ*f* (*a*(*j*)) − Δ*f*(*a*(*i*)) as a predictor of the asymmetry caused by AAU selection.

We will say that two codons are neighbours in the genetic code if they differ by only one of the three positions. In Table 1, *N_SN_*(*i*) is the number of synonymous neighbour codons of any one codon in block *i*. We now define δ*_SN_* (*i,j*) = *N_SN_* (*j*) − *N_SN_* (*i*). According to the translational robustness hypothesis, sequences are selected so that the effects of errors due to codon-anticodon mispairing during translation are minimized. It should, therefore, be preferable to use amino acids with four-codon blocks (*N_SN_*(*i*) = 3) rather than those with two-codon blocks (*N_SN_*(*i*) = 1) because mispairing at the third position has no effect in four-codon blocks. This is the principal effect measured by δ *_SN_*(*i, j*). In four-codon amino acids, both codon blocks have *N_SN_* (*i*) = 3, therefore δ*_SN_* (*i*, *j*) = 0 for synonymous substitutions. Thus δ*_SN_* (*i*, *j*) is principally a predictor of change in amino acid usage, not codon usage. However, for six-codon amino acids (Arg, Leu and Ser), there is also a codon usage effect because the numbers of synonymous neighbours for codons in the three codon blocks are not equal.

A well known effect in molecular evolution is that amino acid substitutions tend to be conservative, i.e. they occur more frequently between amino acids with similar physical properties. Our study of mitochondrial proteins (Urbina et al. 2006) shows that those amino acids that can most easily be replaced by other amino acids with similar physical properties respond most easily to changes in mutation pressure and therefore have the most variable frequencies among species. In particular, the amino acids in the first two columns of the genetic code (with U and C and the second codon position) are much more easily interchangeable than those in the third and fourth columns (with A and G at second position). This observation leads us to another predicted asymmetry effect. If translational robustness is important, codon blocks should be preferred if neighbouring codons code for amino acids with similar physical properties. We will define amino acids as ‘close’ if their physical property distance is less than a threshold value. The definition of the distance measure and the list of pairs of close amino acids are given in detail later in the paper. Let *N_CN_*(*i*) be the number of close neighbours of a codon in block *i*–i.e. the number of codons differing by error at only one position that code for a close amino acid. Synonymous codons are included in this count because an identical amino acid is obviously ‘close’. As translational errors are more frequent at 1st and 3rd position than 2nd, we include only codons that are neighbours by 1st or 3rd position errors in the count of *N_CN_* (*i*). This gives a number between 0 and 6. Hence, we define δ*_CN_* (*i, j*) = *N_CN_* (*j*) − *N_CN_* (*i*) as a predictor of asymmetry. The *N_CN_* (*i*) measure has some similarities with the scoring system used by Archetti (2004) in his study of the influence of protein-level error minimization on codon usage. However, Archetti’s system models mutations, whereas our system models errors in translation, as we intend it to be a predictor of translational robustness. Archetti (2004) includes multiple mutations (up to 10) and specifically makes the point that most synonymous codons would have the same score if only a single mutation were allowed. As we are interested in translational error, we assume that the chance of codon-anti-codon mispairing would be negligible if there were more than one mismatch. Therefore, we only allow for a single position error. Most synonymous codons have the same value of *N_CN_* (*i*), although there are some differences among codons for six-codon amino acids. Thus δ*_CN_*(*i*, *j*), is principally a predictor of asymmetry in amino acid usage, not codon usage, as was also the case with δ*_SN_*(*i*, *j*).

### Simple tests for asymmetry

For each asymmetry effect introduced above, we calculate the number of *ij* pairs, *N_pairs_*, for which δ(*i, j*) > 0. The maximum number of such pairs is 32 × 31/2 = 496, but *N_pairs_* is always less than 496 because pairs where δ(*i, j*) = 0 are excluded. We then count the number of pairs, *N_correct_*, where the sign of Δ*n_ij_* is correctly predicted (both δ(*i, j*) > 0 and Δ*n_ij_* > 0). We calculate the probability *p* that at least *N_correct_* out of *N_pairs_* pairs would have the correct sign if each sign were random. From Table 3, there is significant agreement with the direction of the asymmetry predicted by tRNA, ATP, MW and AAU effects, but no evidence for SN or CN effects. However, it is premature to draw conclusions, because these tests consider each effect alone, and a large effect in one direction might mask a smaller effect in the opposite direction. It is therefore desirable to develop a statistical model in which all these effects can be considered together. Before doing this, we consider two of the asymmetry effects graphically.

**Figure 1.**
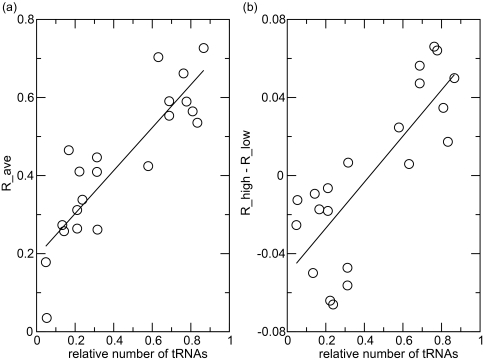
(**a**) Relationship between relative codon frequency and relative number of tRNAs. (**b**) Difference in relative codon frequency between high- and low-expression genes as a function of relative number of tRNAs.

Table 1 gives the frequency π for each codon block (averaged over high- and low-expression genes), and the relative frequency, *R_ave_*, of the codon block with respect to the total frequency of the corresponding amino acid, *f_ave_*. We also calculated relative frequencies *R_high_* and *R_low_* separately in high- and low-expression genes. The difference in these is given in Table 1. Figure 1a shows that *R_ave_* increases as a function of the relative number of tRNAs for the codon block, measured as a fraction of the total number of tRNAs for that amino acid (only cases with more than one codon block per amino acid are considered). This confirms that the average codon usage is influenced by tRNA gene copy number. Figure 1b shows that *R_high_*- *R_low_* also increases with the relative tRNA number. Thus, codon bias is stronger in high-expression than low-expression genes, as expected.

**Figure 2.**
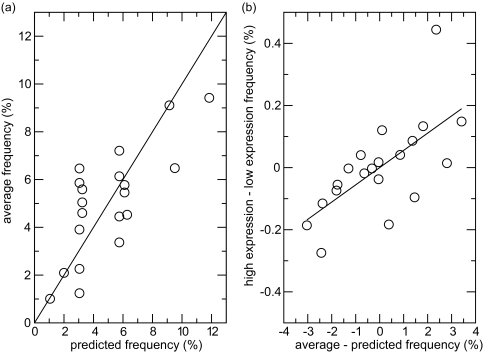
(**a**) Observed average frequency of amino acids versus frequency predicted from GC content. (**b**) Difference in amino acid frequency between high- and low-expression genes as a function of the difference between the average and predicted frequencies.

Figure 2a shows the relationship between *f_ave_* and *f_pred_*. Although there is a positive correlation (*r* = 0.75, p = 1.3 × 10^−4^), there is considerable scatter. Table 2 shows the difference Δ*f* (*a*) between these two frequencies, and also the difference between the observed frequencies in high- and low-expression genes, *f_high_-f_low_*. Figure 2b shows that there is a positive correlation between these two quantities (r = 0.67, p = 1.3 × 10^−3^). Thus, the deviations between observed and predicted frequencies are caused by selection on AAU, and this is stronger in high-expression than low-expression genes.

### Selection of a substitution rate model

Given a model of the data, we can calculate expected frequencies, *f_ij_*, of each pair of states in the alignment. The log-likelihood of the data, given the model, is

$$\text{ln}\;L=\sum_{i}\sum_{j}n_{ij}\text{ln}\;f_{ij}.$$

By definition, AIC = 2(−ln*L̂* + *K*), where *K* is the number of parameters that are estimated from the data, and *L̂* denotes the maximum likelihood value of *L*. The model that best approximates the data is the one with smallest AIC (Burnham and Anderson, 1998). A more complex model with a larger number of parameters will have higher likelihood (−ln*L̂* will be smaller). However, an overly complex model with redundant parameters has a larger *K* but does not significantly decrease −ln*L̂*. AIC selects a model with sufficient parameters, but not too many. The factor of 2 in the definition is conventional, although it makes no difference to the ranking of the models.

Let **r^H^** and **r^L^** be matrices describing the substitution rates in the high- and low-expression genes. The matrices of substitution probabilities in the time *t* since gene duplication are **P^H^**(*t*) = exp(*t***r^H^**) and **P^L^**(*t*) = exp(*t***r^L^**). The pair frequencies *f_ij_* predicted by the model are

$$f_{ij}=\sum_{k}\pi_{k}P_{ki}^{L}(t)P_{kj}^{H}(t),$$

where π*_k_* is the initial frequency of state *k*. We begin with symmetric models, where **r^H^** and **r^L^** are equal to a single time reversible rate matrix **r**. Later we add asymmetric effects.

We distinguish 6 categories of substitutions. Categories 1, 2 and 3 are non-synonymous substitutions requiring 1, 2 and 3 base changes. Category 4 includes synonymous substitutions with a single base change (e.g. Leu(CUR)-Leu(CUY) and Leu(CUR)-Leu(UUR)). Category 5 includes synonymous substitutions requiring 2 base changes where both of these could be synonymous single changes (e.g. Leu(CUY)-Leu(UUR)). Category 6 includes synonymous substitutions requiring 2 or 3 base changes where the individual base changes are non-synonymous (Ser(AGY)-Ser(UCY) and Ser(AGY)-Ser(UCR)).

In the standard symmetric model, S1, six parameters, α_1. . ._ α_6_, control the substitution rates in each category. For synonymous substitutions, *r_ij_* = α_*cat*(*i, j*)_κ*_ij_*π*_j_*, where *cat*(*i,j*) = 4, 5 or 6. For non-synonymous rates, *r_ij_* = α_*cat*(*i, j*)_κ*_ij_*π*_j_* exp(−*d*(*a*(*i*), *a*(*j*))/*D*), where *cat*(*i,j*) = 1, 2 or 3. The diagonal elements are equal to minus the sum of the other elements on the row. For single-substitutioncategories (1 and 4), transitions occur faster than transversions by a factor κ. We define κ*_ij_* = κ for transitions and κ*_ij_* = 1 for transversions. We did not account for transition-transversion rate differences in multiple substitution categories (2, 3, 5 and 6), i.e. κ*_ij_* = 1 for all pairs. Non-synonymous rates follow a decreasing function of the distance *d* between amino acids. *D* is a parameter that controls the shape of this decreasing function. A similar model was used by Goldman and Yang (1994), but only single substitutions were permitted.

An appropriate distance matrix has already been calculated from 8 physical properties of amino acids (Higgs and Attwood, 2005, chapter 2) and has been used to predict evolutionary properties of mitochondrial gene sequences (Urbina et al. 2006). The 8 properties are: 1 = volume (Creighton, 1993); 2 = bulkiness (Zimmerman et al. 1968); 3 = polarity (Zimmerman et al. 1968); 4 = isoelectric point (Zimmerman et al. 1968); 5 = hydrophobicity (Kyte and Doolittle, 1982); 6 = hydrophobicity (alternative scale) (Engelman et al. 1986); 7 = surface area accessible to water (Miller et al. 1987); 8 = fraction of accessible area lost when a protein folds (Rose et al. 1985). Let *z_ak_* be the value of the *k*th property of amino acid *a*, after transforming each property so that its mean is 0 and its standard deviation is 1. The distance *d*(*a*,*b*) is the euclidean distance between amino acids *a* and *b* in the 8-dimensional *z-*space (Urbina et al. 2006).

There are 32 frequencies π*_i_* that must add up to 1; hence 31 independent parameters. We set these to the observed average frequencies of the states (Table 1). These parameters are estimated from the data; therefore they contribute 31 towards *K* in the AIC equation. As the total amount of data is large (*n_tot_* = 146398), the observed frequencies will be very close to the ML frequencies. These 31 parameters are treated equivalently in all models and therefore they do not affect the ranking by AIC. We expect α_4_ to be the largest of the rate parameters. Therefore we set α_4_ = 1, and measure the other 5 rate parameters relative to this. Finally, rates are normalized such that the mean substitution rate is equal to 1. This sets the time scale such that *t* is the mean number of substitutions per site.

There are 8 parameters in S1 for which ML values must be estimated from the data (5 α values, *t*, κ, and *D*). Hence, the total number of parameters is *K* = 39 for S1. The 8 ML values were found by a random hill-climbing routine beginning from an initial rough estimate. At each iteration, one random parameter was changed by a small random amount, and the new parameter was accepted if ln *L* increased. 30000 iterations of this process were sufficient to give good convergence for the S series of models (at least 3 significant figures in parameter values and less than 0.01 in ln*L*). For the W and A series models, 80000 iterations were used. Parameters were found to converge to the same values from several different initial points. No problems of local optima were encountered.

Optimal parameters for model S1 are given in Table 4. The rate parameters rank in the order α_4_ > α_5_ > α_6_ > α_1_ > α_2_ > α_3_, which we would expect if synonymous substitutions are faster than non-synonymous ones, and if single base changes are faster than double and triple changes. Models S2–S8 are variants that test the assumptions of model S1. Model S2 removes the difference between transitions and transversions by setting κ = 1. This leads to a large increase in AIC. In Table 5, ΔAIC is the difference in AIC with respect to S1. When interpreting ΔAIC values, it should be remembered that the relative weight to be associated with a model is proportional to exp(−ΔAIC/2)–see Burnham and Anderson (1998), section 2.9. Most of the ΔAICs in Table 5 are very large, so that the relative weights of the less well fitting models are very small compared with the better models. A rule of thumb is that models with ΔAIC < 2 have considerable support as alternatives, those with 2 < ΔAIC < 10 have weak support, and those with ΔAIC > 10 have essentially no support. Model S2 is thus rejected with respect to S1, which confirms that transitions are faster than transversions.

In models S3, S4, and S5 some of the α parameters for multiple substitutions are set to zero. All of these lead to large increases in AIC and are thus rejected. Thus, the data are not well described by a model that allows only single base changes at a time. Models S6, S7 and S8 consider variations in the way that the rates depend on amino acid distance. In S6, there is no distance function, i.e. *r_ij_* = α_*cat*(*i, j*)_κ*_ij_*π*_j_* for the non-synonymous changes as well as the synonymous ones. S7 uses a gaussian distance function *r_ij_* = α_*cat*(*i, j*)_κ*_ij_*π*_j_* exp(−(*d*(*a*(*i*), *a*(*j*))/*D*)^2^) for the non-synonymous rates, and S8 uses a power law function *r_ij_* = α_*cat*(*i, j*)_κ*_ij_*π*_j_*/*d*(*a*(*i*), *a*(*j*))^β^, where β is a parameter to be estimated. According to AIC, S7 and S8 are both much worse than S1 but much better than S6. This suggests that rates do decrease as a function of amino acid distance and that the exponential function models this effect better than the gaussian or the power law.

Since the amino acid distance is an important factor in the model, it is worth asking if the distance measure itself can be improved. The 8 properties used may not be equally important. We therefore assigned variable weights *w_k_* to the properties. We also added a 9th property that was not included by Higgs and Attwood (2005). This is the polar requirement scale (Woese et al. 1966), which has been shown to be important in studies on the genetic code (Haig and Hurst, 1991; Freeland and Hurst, 1998), and which is likely to be a property that influences substitution rates. We define a weighted distance measure

$$d(a,b)={\left(\sum_{k=1}^{9}w_{k}{\left(z_{ak}-z_{bk}\right)}^{2}\right)}^{1/2},$$

with the constraint that the 9 weights sum to 1, so that there are 8 independent weight parameters. Model W1 is equivalent to S1 except that the *w_k_* are additional parameters. These are estimated by the same numerical optimization technique, starting with an initial state where all properties are equally weighted.

Model W1 is strongly preferred over model S1. The difference in AIC between these two models is 4330.6. The optimal weights for model W1 are given in Table 4. Properties 1, 3, and 6 have weights that tend to 0 during optimization. In hindsight, we could have eliminated these three properties from the model, in which case the AIC would be reduced by 6. However, we had no *a priori* reason why these particular three should be eliminated, and to remove them at this point would amount to ‘data dredging’, in the sense of (Burnham and Anderson, 1998). The W and A models considered below are variants on W1. In each of these models, all 9 weights were included, but the same three weights converged to 0 in the optimization. For each of these models, the weights were counted as 8 parameters for the AIC. The three redundant parameters make no difference to the ranking of these models.

Another factor that often improves the fit of data used for molecular phylogenetics is to allow variation in rates across sites. We added this using a number of discrete Γ-distributed rate categories (Yang, 1994). This involves addition of one extra parameter, λ, that controls the shape of the Γ distribution. Models W2, W3 and W4 are equivalent to W1 with the addition of 2, 3, and 4 rate categories. All these are improvements over W1. W3 is the best of the W series and is the reference for ΔAIC in Table 5. As W3 is the best symmetric model, the distance matrix derived from the ML weights in W3 is the most meaningful scale of distance between amino acids. Distances between typical amino acids are close to 1 on this scale. Amino acids with *d* < 0.8 were counted as close pairs in the calculation of *N_CN_*. These are FL, FI, FM, FV, FW, LI, LM, LV, IM, IV, MV, MY, SP, ST, SA, SN, SG, PT, PQ, PN, TA, YW, HQ, HN, QN, QK, QE, ND, NE, KR and DE.

Model W5 is equivalent to W3 with κ set to 1. Models W6, W7, and W8 are variants in which some of the rates of multiple substitutions are set to 0. All of these models perform much worse than W3, according to AIC. Thus, we retain W3 as the best symmetric model, and we consider the effects of asymmetry in substitution rate by introducing perturbations to this model.

### Is there asymmetry in the substitution rate?

Let *r_ij_* be the rate matrix for W1. From this we can define distinct rate matrices for the high- and low-expression lineages as follows. For synonymous substitutions,

$$\begin{array}{l} r_{ij}^{H}=r_{ij}(1+{ɛ}_{tRNA}\delta_{tRNA}(i,j)+{ɛ}_{SN}\delta_{SN}(i,j)\\ \;\;\;\;\;\;\;\;\;\;\;\;+{ɛ}_{CN}\delta_{CN}(i,j)), \end{array}$$

and for non–synonymous substitutions,

$$\begin{array}{l} r_{ij}^{H}=r_{ij}(1+{ɛ}_{tRNA-NS}\delta_{tRNA}(i,\;j)+{ɛ}_{ATP}\delta_{ATP}(i,j)\\ \;\;\;\;\;\;\;\;\;\;\;\;+{ɛ}_{MW}\delta_{MW}(i,j)+{ɛ}_{AAU}\delta_{AAU}(i,j)\\ \;\;\;\;\;\;\;\;\;\;\;\;+{ɛ}_{SN}\delta_{SN}(i,j)+{ɛ}_{CN}\delta_{CN}(i,j)), \end{array}$$

where the δ’s are the asymmetry factors defined above, and the ɛ’s are model parameters that quantify the strength of the effect. The principle is that positive values of the δ’s should correlate with increased rates of substitution on the branch to the high-expression genes. On the branch to the low-expression genes, these effects are reversed; hence $r_{ij}^{L}$ is defined in the same way except that there is a negative sign in front of all the ɛδ terms. The values of the ɛ parameters must be ≥0. Negative values are not permitted by the optimization routine. Note that translational efficiency selection could influence both synonymous and non-synonymous substitutions, but its effect is likely to be strongest on synonymous substitutions. Therefore we introduced parameters ɛ*_tRNA−NS_* in the non-synonymous rates and ɛ*_tRNA_* in the synonymous rates that are optimized separately. The full asymmetric model, A8, includes all 7 ɛ parameters. The other A-series models include subsets of the ɛ’s (Table 6). All these models include 3 Γ distributed rate categories, i.e. they reduce to W3 if all the ɛ’s are zero.

Models A1–A7 consider the asymmetry effects separately. A1 includes only the tRNA effect in the synonymous rates. This leads to a large reduction in AIC with respect to W3 (see ΔAIC* column in Table 6). A2 includes tRNA effects in both synonymous and non-synonymous rates. During parameter fitting, ɛ*_tRNA−NS_* converged to 0; hence the solution is the same as model A1. There is thus no evidence for a tRNA effect on non-synonymous substitutions, even though the effect on synonymous substitutions is large. Models A3, A4 and A5 consider ATP, MW and AUU effects individually. Each of these gives a noticeable improvement in AIC with respect to W3. Models A6 and A7 consider the two measures of translational robustness. In A6, ɛ*_SN_* converges to 0, and the likelihood is the same as W3, whereas in A7, there is some improvement in AIC due to the addition of ɛ*_CN_*.

When all 7 asymmetry factors are added simultaneously (A8), the optimum solution has non-zero values for ɛ*_tRNA_*, ɛ*_AAU_*, ɛ*_SN_* and ɛ*_CN_*, but the other 3 ɛ’s converge to zero. Model A9 includes only the four non-redundant asymmetry effects. It therefore has the same likelihood as A8 and an improved AIC because it has 3 fewer parameters. A9 is not quite the best model, because if ɛ*_CN_* is also eliminated, leaving only ɛ*_tRNA_*, ɛ*_AAU_* and ɛ*_SN_* (A10), this leads to a slight reduction in AIC. If ɛ*_CN_* is included instead of ɛ*_SN_* (A11), the result is substantially worse than either A9 or A10, and if neither ɛ*_SN_* or ɛ*_CN_* is included (A12), the result is worse again. In summary, if tRNA and AAU effects are already accounted for, then addition of either ɛ*_SN_* or ɛ*_CN_* improves the fit. Translational robustness seems to be better modelled by ɛ*_SN_* than ɛ*_CN_*. If ɛ*_SN_* is already included, then addition of ɛ*_CN_* has only a marginal effect. This is different from the conclusion when considering these effects singly (A6 and A7).

Thus, the model selected by AIC (A10) includes ɛ*_tRNA_*, ɛ*_AAU_* and ɛ*_SN_*. Models A12, A13 and A14 establish that if any one of these three parameters is eliminated, the quality of fit to data is significantly worse. It is surprising that the ATP and MW effects should drop out, given that these are important when considered alone (A3, A4). There appears to be some redundancy of these factors with AAU. In model A15, we included ATP and MW effects alongside tRNA and SN effects, but excluded AAU. In this case, both ɛ*_ATP_* and ɛ*_MW_* were non-zero in the optimal solution. This gives an improvement with respect to A13 (i.e. there is some benefit from inclusion of ATP and MW), but it is substantially worse than A10.

Inclusion of any one of the asymmetry effects is sufficient to mean that *f_ij_* ≠ *f_ji_* for every pair. For each A model, there are 496 pairs for which *f_ij_* > *f_ji_*. Table 6 shows *N_correct_*, the number of these pairs for which Δ*n_ij_* > 0, and the *p* values from the sign test. These may be compared with Table 3. Models with better AIC scores tend also to have larger *N_correct_*.

## Discussion and Conclusions

As simultaneous multiple mutations within one codon are likely to be rare, we might expect that a mutation matrix that permits only single base changes would be sufficient. In fact, this was not the case. This was shown initially with models S3–S5, which assume all sites evolve at constant rates. We might worry that apparent double substitutions are an artefact resulting from two separate single substitutions happening at a fast evolving site in the time that a slowly evolving site changes only once. Nevertheless, the same result is seen with models W6–W8, which account for variation in rates across sites. We have pointed out the same effect in the paired regions of RNA secondary structure (Higgs, 2000; Savill et al. 2001). This can be explained by compensatory mutation theory (Higgs, 1998), because each single mutation is likely to be deleterious, but the two together may be close to neutral. It is possible that the same thing is occurring in protein sequences, e.g. between the two families of Ser codons (parameter α_6_). It is also possible that there is some specific mutational process that operates on groups of successive bases. In amino acid substitution matrices, such as PAM (Jones et al. 1992), all possible substitutions are allowed without considering the number of base changes. However, in most codon-based models (Goldman and Yang, 1994; Yang et al. 2000; Pond and Muse, 2005), it is assumed that only single substitutions are allowed. Our results suggest that this is not optimal. Whelan and Goldman (2004) consider a singlet-doublet-triplet (SDT) model that allows both doublet and triplet substitution events in addition to single changes, and find that these occur at an appreciable rate. The SDT model allows overlapping, out-of-frame doublets and triplets, whereas our models above consider independent codons. However, the SDT model does not consider the effect of the amino acid properties, which is included in our model. Whelan and Goldman (2004) also point out that doublet and triplet changes can arise either from a mutational event that affects multiple bases (like gene conversion) or from compensatory substitutions. Whatever the explanation for the multiple substitutions seen here, it should be remembered that the rate matrices do not describe the rate of mutations in individual genes but rather the rate of fixation of new variant sequences in the population. Simultaneous fixation of two substitutions does not imply that two mutations occurred simultaneously.

The predicted amino acid frequencies in Figure. 2 take account of the observed GC content. If the GC content were 0.5, the predicted frequencies would be proportional to the quota of codons for each amino acid in the genetic code. There is a positive correlation of *f_ave_* with the codon quotas (r = 0.62), but this is less strong than when the true GC content is used (r = 0.75). There may have been some degree of optimization of the quotas in the code to meet the amino acid requirements of proteins (Dufton, 1997). However, if such an optimization occurred as the canonical code was being established, it could not be dependent on variation in GC between organisms, or on gene expression level. In the data studied here, amino acid frequencies are influenced by both the codon quotas and the GC content, but also, more importantly, by AAU selection that acts on top of these ‘passive’ effects.

The AAU effect is not independent of the ATP and MW effects because the cost of amino acid synthesis is one factor that could drive AAU selection (i.e. less costly amino acids might have positive Δ*f*). In fact, there is a strong correlation between *N_ATP_* and *MW* (r = 0.80, p = 2.0 × 10^−5^), and a weak negative correlation between *N_ATP_* and Δ*f* (r = −0.47, p = 0.035) and between *MW* and Δ*f* (r = −0.33, p = 0.15). However, AAU will be driven by the amino acid requirements for protein function, which might bear little relationship to costs of synthesis, and might be the aggregate of many different selective effects. Therefore, the AAU hypothesis is much more general than the ATP and MW hypotheses. Our interpretation of the result that the ATP and MW effects drop out of the best model is that selection for cost minimization is subsumed within the more general measure of AAU selection. Although Δ*f* is useful as a measure of AAU selection, it has the drawback that it does not explain why amino acids are positively or negatively selected.

These results give support to the translational robustness hypothesis. The AIC method provides a means of locating the translational robustness effect in the presence of larger, potentially conflicting factors, even though it does not show up at all in the simple tests in Table 3. The importance of an appropriate choice of statistical method to deal with multiple effects was also emphasized in Drummond et al. (2006), where it was concluded that expression level, codon adaptation index, and protein abundance are the key variables determining the variation of rate of evolution between genes. This was attributed to ‘translational selection’, although in the treatment of Drummond et al. (2006), it is not clear whether this is efficiency or robustness. The difference between these is clear in Drummond et al. (2005) and also in our own analysis above. Our main conclusion is that the most important factors causing asymmetry between high- and low-expression genes are translational efficiency selection on synonymous substitutions, and selection on amino acid usage. Having accounted for these, a further small effect of translational robustness is found.

## Figures and Tables

**Table 1.** Codon group properties.

**Codon group**	***N*_tRNA_**	π	***R*_ave_**	***R*_high_*-R*_low_**	***N*_SN_**	***N*_CN_**
Phe(UUY)	10	4.455	1	0	1	6
Leu(UUR)	17	5.314	0.564	0.035	2	6
Leu(CUY)	1	1.682	0.179	−0.025	3	6
Leu(CUR)	3	2.425	0.257	−0.009	4	6
Ile(AUY)	13	4.705	0.727	0.050	2	6
Ile(AUA)	2	1.177	0.273	−0.050	2	6
Met(AUG)	5	2.094	1	0	0	6
Val(GUY)	14	3.217	0.589	0.064	3	6
Val(GUR)	4	2.241	0.411	−0.064	3	6
Ser(UCY)	11	3.860	0.424	0.025	3	6
Ser(UCR)	4	2.840	0.312	−0.018	3	6
Ser(AGY)	4	2.404	0.264	−0.006	1	2
Pro(CCY)	2	2.139	0.465	−0.017	3	5
Pro(CCR)	10	2.464	0.535	0.017	3	5
Thr(ACY)	11	3.196	0.553	0.047	3	6
Thr(ACR)	5	2.579	0.447	−0.047	3	6
Ala(GCY)	11	3.305	0.592	0.056	3	5
Ala(GCR)	5	2.281	0.408	−0.056	3	5
Tyr(UAY)	8	3.371	1	0	1	1
His(CAY)	7	2.263	1	0	1	4
Gln(CAR)	9	3.906	1	0	1	5
Asn(AAY)	10	6.139	1	0	1	2
Lys(AAR)	21	7.208	1	0	1	2
Asp(GAY)	15	5.858	1	0	1	4
Glu(GAR)	16	6.467	1	0	1	4
Cys(UGY)	4	1.243	1	0	1	1
Trp(UGG)	6	1.007	1	0	0	0
Arg(CGY)	6	1.183	0.261	0.007	3	3
Arg(CGR)	1	0.160	0.035	−0.013	4	4
Arg(AGR)	12	3.181	0.703	0.006	2	2
Gly(GGY)	16	3.341	0.662	0.066	3	4
Gly(GGR)	5	1.704	0.338	−0.066	3	3

**Table 2.** Amino acid properties.

**Amino acid**	***N*_ATP_**	***MW***	***f*_pred_**	***f*_ave_**	Δ***f***	***f*_high_*-f*_low_**
Phe	52.0	165	5.754	4.455	−1.299	−0.003
Leu	27.3	131	11.852	9.420	−2.432	−0.275
Ile	32.3	131	9.514	6.476	−3.038	−0.186
Met	34.3	149	1.993	2.094	0.101	0.120
Val	23.3	117	6.099	5.458	−0.641	−0.018
Ser	11.7	105	9.148	9.104	−0.044	−0.038
Pro	20.3	115	3.232	4.603	1.370	0.086
Thr	18.7	119	6.099	5.775	−0.324	−0.003
Ala	11.7	89	3.232	5.585	2.353	0.444
Tyr	50.0	181	5.754	3.371	−2.382	−0.115
His	38.3	155	3.049	2.263	−0.786	0.040
Gln	16.3	146	3.049	3.906	0.856	0.041
Asn	14.7	132	5.754	6.139	0.385	−0.183
Lys	30.3	146	5.754	7.208	1.455	−0.096
Asp	12.7	133	3.049	5.858	2.808	0.014
Glu	15.3	147	3.049	6.467	3.418	0.148
Cys	24.7	121	3.049	1.243	−1.807	−0.074
Trp	74.3	204	1.056	1.007	−0.050	0.017
Arg	27.3	174	6.282	4.524	−1.758	−0.055
Gly	11.7	75	3.232	5.045	1.813	0.133

**Table 3.** Test of individual asymmetry effects.

	***N*_pairs_**	***N*_correct_**	**% correct**	***p***
tRNA	474	291	61	4.0 × 10^−7^
ATP	456	256	56	5.0 × 10^−3^
MW	474	266	56	4.4 × 10^−3^
AAU	481	283	59	6.2 × 10^−5^
SN	352	165	47	0.89
CN	390	180	46	0.94

**Table 4.** ML parameters for the most important models.

S1	α_1_ = 0.0691; α_2_ = 0.0282; α_3_ = 0.0238; α_4_ = 1; α_5_ = 0.396; α_6_ = 0.121;
	*t* = 0.757; *D* = 3.407; κ = 1.707.

W1	α_1_ = 0.0850; α_2_ = 0.0405; α_3_ = 0.0450; α_4_ = 1; α_5_ = 0.375; α_6_ = 0.118;
	*t* = 0.735; *D* = 0.901; κ = 1.593;
	*w*_1_ = 0; *w*_2_ = 0.151; *w*_3_ = 0; *w*_4_ = 0.021; *w*_5_ = 0.226; *w*_6_ = 0; *w*_7_ = 0.265; *w*_8_ = 0.184; *w*_9_ = 0.154.

W3	α_1_ = 0.0558; α_2_ = 0.0198; α_3_ = 0.0264; α_4_ = 1; α_5_ = 0.274; α_6_ = 0.0784;
	*t* = 1.345; *D* = 0.800; κ = 1.698; λ = 1.812;
	*w*_1_ = 0; *w*_2_ = 0.155; *w*_3_ = 0; *w*_4_ = 0.028; *w*_5_ = 0.218; *w*_6_ = 0; *w*_7_ = 0.277; *w*_8_ = 0.179; *w*_9_ = 0.142.

A10	α_1_ = 0.0561; α_2_ = 0.0199; α_3_ = 0.0265; α_4_ = 1; α_5_ = 0.274; α_6_ = 0.0785;
	*t* = 1.346; *D* = 0.799; κ = 1.696; λ = 1.511;
	*w*_1_ = 0; *w*_2_ = 0.155; *w*_3_ = 0; *w*_4_ = 0.028; *w*_5_ = 0.218; *w*_6_ = 0; *w*_7_ = 0.278; *w*_8_ = 0.179; *w*_9_ = 0.142;
	ɛ_tRNA_ = 6.509 × 10^−3^; ɛ_AAU_ = 1.191; ɛ_SN_ = 0.0111.

**Table 5.** Model selection criteria for the symmetric models. Δ*AIC* is measured relative to the best model in each group.

	*−****ln L***	***K***	Δ***AIC***	**Notes**
S1	836619.3	39	0.0	Standard Symmetric Model
S2	837192.4	38	1144.2	κ = 1
S3	837649.2	38	2057.8	α_3_ = 0
S4	848644.1	37	24045.6	α_2_ = 0 and α_3_ = 0
S5	838036.5	38	2832.4	α_6_ = 0
S6	841074.9	38	8909.2	No distance function
S7	836991.9	39	745.2	Gaussian distance function
S8	837205.5	39	1172.4	Power law distance function

W1	834446.0	47	139.8	Weighted Distance Model
W2	834384.2	48	18.2	2Γ
W3	834375.1	48	0.0	3Γ
W4	834376.9	48	3.6	4Γ
W5	834780.7	47	809.2	3Γ, κ = 1
W6	835088.8	47	1425.4	3Γ, α_3_ = 0
W7	837763.2	46	6772.2	3Γ, α_2_ = 0 and α_3_ = 0
W8	835454.3	47	2156.4	3Γ, α_6_ = 0

**Table 6.** Model selection criteria for the asymmetric models. Δ*AIC* is measured relative to A10. ΔAIC* is measured relative to W3.

	***ln L***	***K***	Δ***AIC***	ΔAIC*	***N*_correct_**	***p***	**Effects included**
A1	834224.5	49	93.0	−299.2	295	1.4 × 10^−5^	tRNA
A2	834224.5	50	95.0	−297.2	295	1.4 × 10^−5^	tRNA, tRNA−NS
A3	834367.9	49	379.8	−12.4	276	6.7 × 10^−3^	ATP
A4	834364.2	49	372.4	−19.8	276	6.7 × 10^−3^	MW
A5	834340.8	49	325.6	−66.6	310	1.4 × 10^−8^	AAU
A6	834375.1	49	394.2	2.0	232	0.93	SN
A7	834367.9	49	379.8	−12.4	238	0.83	CN

A8	834175.8	55	7.6	−384.6	315	9.4 × 10^−10^	Full Asymmetric Model
A9	834175.8	52	1.6	−390.6	315	9.4 × 10^−10^	tRNA, AAU, SN, CN
A10	834176.0	51	0.0	−392.2	315	9.4 × 10^−10^	tRNA, AAU, SN
A11	834184.7	51	17.4	−374.8	318	1.7 × 10^−10^	tRNA, AAU, CN
A12	834189.8	50	25.6	−366.6	324	4.2 × 10^−12^	tRNA, AAU
A13	834216.6	50	79.2	−313.0	299	2.7 × 10^−6^	tRNA, SN
A14	834340.8	50	327.6	−64.6	309	2.4 × 10^−8^	AAU, SN
A15	834211.4	52	72.8	−319.4	314	1.6 × 10^−9^	tRNA, ATP, MW, SN
